# Supplementary figures and images for: LimoRhyde2: Genomic analysis of biological rhythms based on effect sizes
Source: PLoS One. 2023 Dec 14;18(12):e0292089. doi: 10.1371/journal.pone.0292089 (PMC10721038; doi:10.1371/journal.pone.0292089)

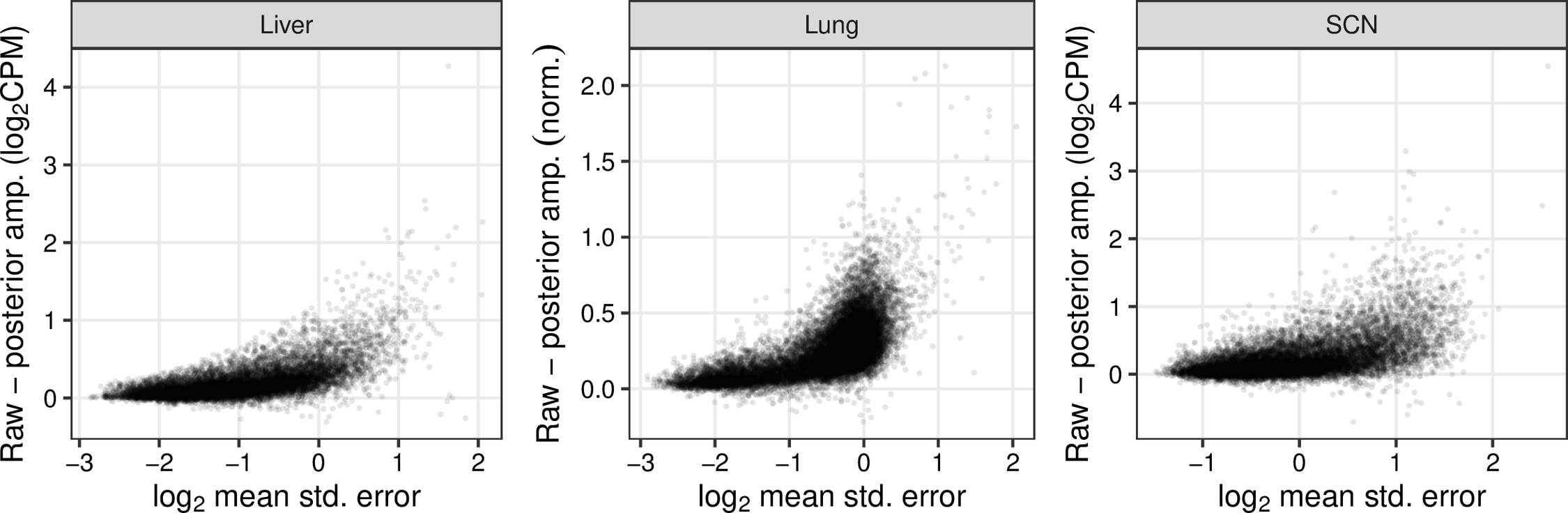

Supplement: S1 Fig — Scatterplots of difference between raw and posterior peak-to-trough amplitude vs log2 mean standard error of the raw fit for genes in each tissue. Points represent genes. (TIF) [file pone.0292089.s001.tif]

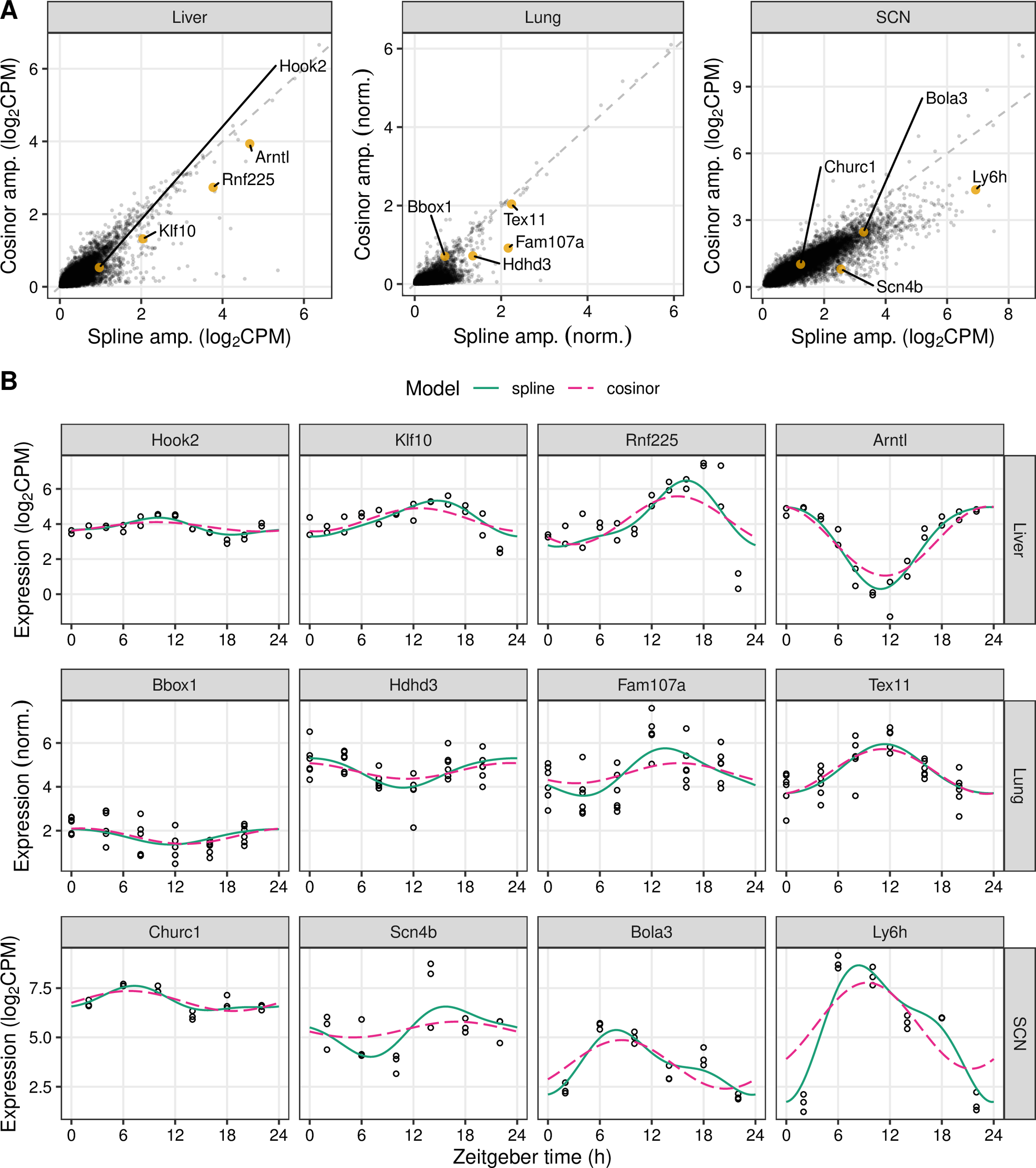

Supplement: S2 Fig — (A) Scatterplots of cosinor posterior peak-to-trough amplitude vs. spline posterior peak-to-trough amplitude for each tissue (indicated at top). Points represent genes. Dashed lines indicate y = x. (B) Time-courses of expression of genes labeled in (A) in the respective tissue (indicated at right). Points represent samples. Curves represent posterior fits for the two models. (TIF) [file pone.0292089.s002.tif]

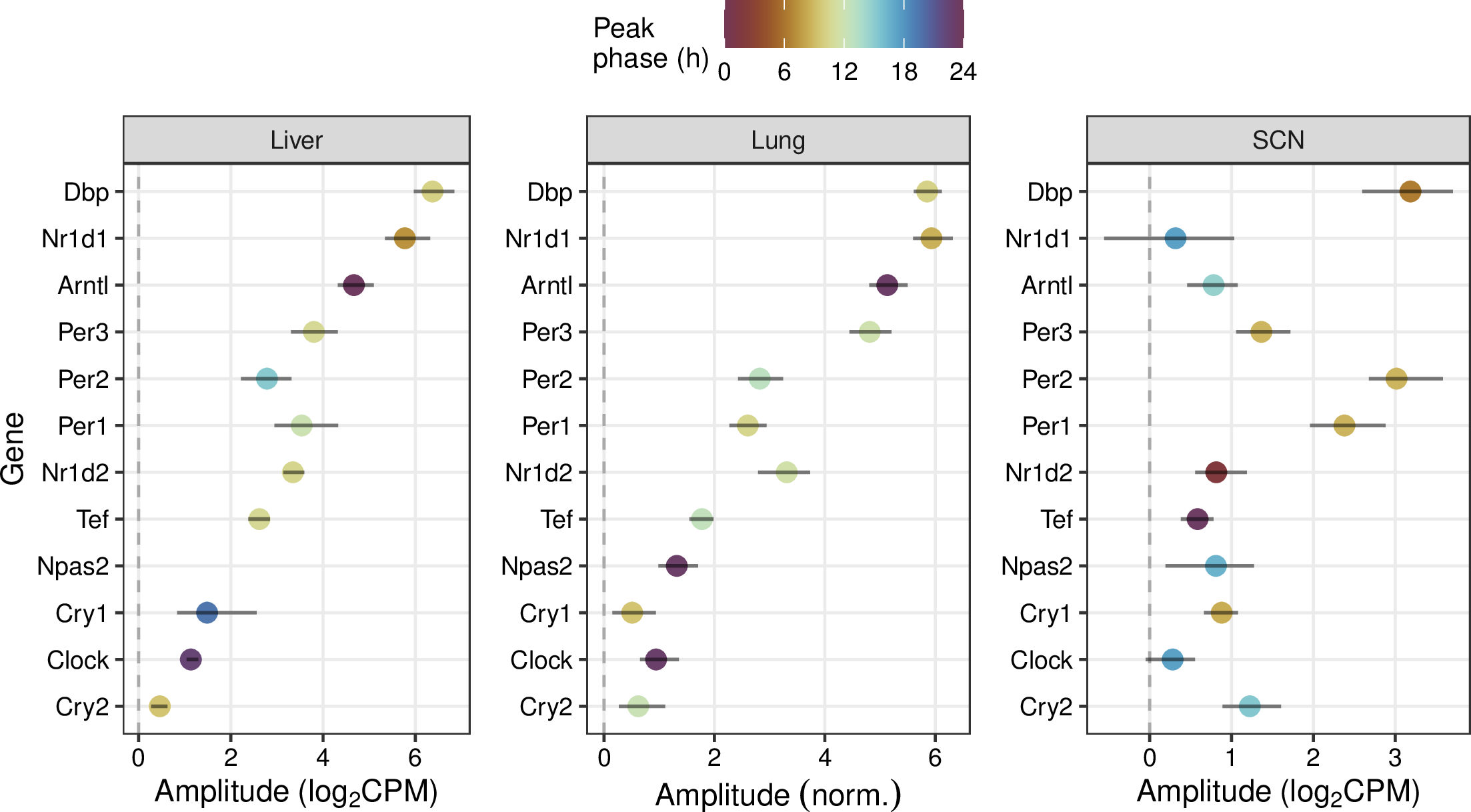

Supplement: S3 Fig — Posterior peak-to-trough amplitudes and corresponding 90% credible intervals for core clock genes in each tissue. Points represent genes, color represents peak phase for each gene. Dashed lines indicate 0 amplitude. (TIF) [file pone.0292089.s003.tif]

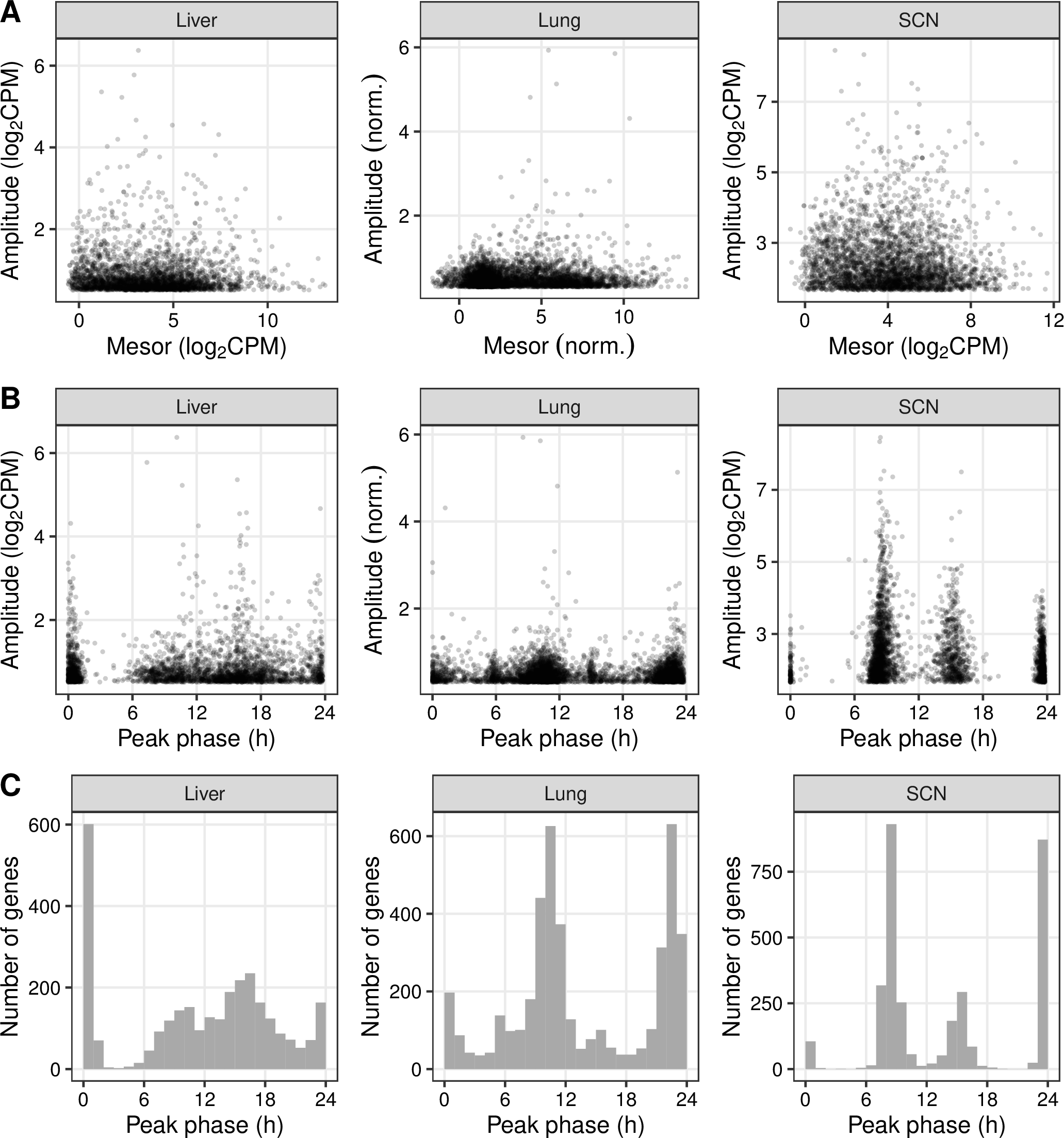

Supplement: S4 Fig — Scatterplots of (A) peak-to-trough amplitude vs. mesor and (B) peak-to-trough amplitude vs. peak phase for genes in each tissue (indicated at top). Points represent genes. (C) Histograms of peak phase. All plots include only the top 25% of genes based on amplitude. (TIF) [file pone.0292089.s004.tif]
