# Supplementary material for: LimoRhyde2: Genomic analysis of biological rhythms based on effect sizes
Source: PLoS One. 2023 Dec 14;18(12):e0292089. doi: 10.1371/journal.pone.0292089 (PMC10721038; doi:10.1371/journal.pone.0292089)
Supplement: Attachment — Submitted filename: 133934_1_rebuttal_2689288_rv9q6k.docx [file pone.0292089.s005.docx]

**Manuscript number:** RC-2023-01863

**Corresponding author:** Jacob Hughey

## 1. General Statements

We thank the reviewers for their constructive feedback, which has helped us improve the manuscript considerably (no comment on whether the improvements are “significant”). Below are our point-by-point responses. We have also highlighted all changes in the manuscript.

## 2. Point-by-point description of the revisions

### Reviewer 1

#### Summary

In this study, Obodo et al. present a new iteration of their popular rhythm analysis tool LimoRhyde. The conceptual advancement in this new iteration is the focus on effect sizes (in the form of point estimates of amplitude and their prediction intervals) rather than the p-values, which has been the predominant form of statistical testing for rhythm analysis. Therefore, compared to a well-established non-parametric method for rhythm testing, LimoRhyde2 selects genomic features with larger amplitudes (effect-sizes) as it is designed to do.

#### Major Comments

1. (LimoRhyde2 algorithm, Page 2-) It is unclear what exactly the contributions/advancements of the authors are? Is it a novel statistical method, the combination of well-established tools in a novel workflow, or is it a novel application to a new field (rhythms)? I am afraid the sentence "LimoRhyde2 builds on previous work by our group and others to rigorously analyze data from genomic experiments [9,16,17], capture non-sinusoidal rhythms [18], and accurately estimate effect sizes [14,19]." is rather ambiguous.

We have revised this sentence in the last paragraph of the Introduction to clarify LimoRhyde2’s contributions.

2. (Moderate model coefficients, Page 3-) The authors implement empirical Bayes shrinkage on the coefficients. But the state-of-the-art methods used in LimoRhyde2 for linear model fitting, such as DESeq2/limma-voom/limma-trend, already implement shrinkage for the coefficients. Does algorithm implement a second round of Bayes shrinkage on the rhythm effect-sizes? How or why is this a statistically valid procedure? If not, how does Limorhyde2 add to shrinkage already implemented in DESeq2/limma-voom/limma-trend? Please elaborate.

To our understanding, the two shrinkage procedures work at different levels and serve different purposes. Limma applies shrinkage on residual variances to account for any technical variation and to give a higher power to detect effects for data with smaller sample sizes within each condition; it does not shrink coefficients. In practice, limma’s shrinkage has little effect given the relatively large sample sizes of most circadian experiments. LimoRhyde2, on the other hand, uses mashr to apply shrinkage to the coefficients themselves to account for shared patterns of effects and variation across both features and conditions. We see no reason this approach is invalid, and in our conversations with Matthew Stephens, the author of ashr and mashr, he felt the same. We elaborate on each method’s contributions in the Discussion (paragraph 2).

3. I think the goal to move to effect-sizes which lead to more reproducible results and better biological significance is sound and highly appreciated. However, to make the community switch to a completely different way of viewing their genomic analysis requires more convincing examples(s)/use-cases on why they should abandon the old method that they are used to. Now, results section merely shows that this algorithm performs as designed (to find large amplitude rhythms).

We appreciate the comment and acknowledge that some readers may be particularly attached to p-values and our current analysis may not wholly convince them of the value of effect sizes. We believe the manuscript stands on its own, however, and are using LimoRhyde2 to guide experiments whose conclusions we hope to describe in future work. Nonetheless, we have revised the Discussion (paragraph 4) to clarify that some known relevant genes highly ranked by LimoRhyde2 were underappreciated by BooteJTK.

4. Related to point 3, others have previously proposed using amplitude (effect-size) thresholds in addition to the p-value cutoffs (Lück & Westermark, 2016, Pelikan et al, 2022), how would the results of Limorhyde2 compare in a fairer contrast where both p-value and amplitude thresholds are implemented? Does the proposed sound method outperform the two-step approach. The authors may perform this analysis on their chosen datasets as well.

Thank you for raising this point. Indeed, one way to view LimoRhyde2 is as a data-driven balancing of raw effect size and p-value. However, the approach of considering both raw amplitude and p-value is uncommon and requires yet another arbitrary cutoff, which complicates any genewise ranking and side-by-side comparison with other methods. Thus, we have decided to not perform this analysis, and instead mention what we see as the advantage of LimoRhyde2 in the Discussion (paragraph 2).

5. I am also not completely convinced of the author's approach to compare their tool against BooteJTK. P-values only show ordering when the alternative hypothesis is true. P-values under the null hypothesis are uniformly distributed in [0,1] so would be meaningless for the purpose of ordering. Without knowing the ground-truth, ordering by p-values is rather risky. I understand the authors' difficulty. But maybe point 4 above yields a better evaluation strategy for LimoRhyde2.

If one accepts that these datasets have a non-zero number of “true” rhythmic genes, which to us seems more than reasonable, then we don’t see this is a large issue. Ranking by (adjusted) p-value is also the standard in differential expression analyses.

6. (OPTIONAL) LimoRhyde2 orders results by the point estimates of the effect-sizes (amplitudes). Is this biologically the most meaningful? Should the effect-size CIs be ordered at all? Maybe we only care about whether the lower limit of the CI is greater than a chosen threshold without any ordering. A discussion of this would be valuable to a user.

We discussed this issue amongst ourselves as well, and ultimately elected for simplicity in ranking by only the point estimate and not the credible interval. We have now mentioned this issue in the penultimate paragraph of the Discussion.

7. (OPTIONAL) If indeed the authors want to move away from p-values, one could argue that most of the insights from p-value analysis are or could be biased. So why compare against ordering by p-values at all in the results?

We are not arguing that results from p-value-based analyses are biased. We seek to show the differences on real data between an analysis based on p-values, the dominant approach in the field, and one based on estimated effect sizes. We believe this has greater potential to promote thoughtful progress than does outright rejection of p-values based on a purely theoretical argument.

#### Minor Comments

1. In page 3, it is unclear why averaging the three fits is the best thing to do? How bad would the performance be if m = 1 was chosen compared to m=3.

We have elaborated the relevant section of the Methods. For most genes in most datasets, the difference between m=1 and m=3 wasn’t much. However, m=1 tended to go noticeably sideways for some of the most rhythmic genes, depending on the relative locations of timepoints and spline knots, whereas m=3 did not.

2. In page 4, "To account for this uncertainty, LimoRhyde2 constructs..." was difficult to understand and sounded arbitrary. Please explain further.

We have revised this sentence.

3. Lachmann et al. (2021) also use bootstrap confidence intervals rather than p-values to quantify rhythmicity that ought to be mentioned.

We have now cited this paper in the Introduction.

#### Significance Comments

1. General assessment: The authors present an exciting new way of viewing results of high-throughput data analysis in the context of biological rhythms using a Bayesian-like approach. Previously work has revealed the flaws in focusing on p-values and how focusing of effect-sizes (in this context amplitudes) can yield more robust, reproducible results. Although this promises to also yield more biological meaningful results, it is unclear from this study how this might be.

See reply to Major Comment 3 above.

2. Advance: This study presents the first tool in the context of the rhythm analysis to provide prediction intervals for different rhythm parameters to facilitate a move away from the hypothesis testing framework of p-values. This is a technical advance in the field of rhythm analysis, but it is unclear what insights this could yield.

See reply to Major Comment 6 above.

### Reviewer 2

#### Major Comments

1. The manuscript introduces a new tool to select rhythmic genes and to quantify amplitudes and phases. The authors combine splines, linear regression, Bayes sampling, and Mash. They focus on amplitudes instead p-values as in other packages. The performance and independence of JTK methods are illustrated using selected circadian expression profiles from different mammalian tissues. The paper is clearly written and provides a valuable extension of existing tools. I miss, however, an intuitive explanation of Mash.

Thank you.

2. I agree with their claim that amplitudes are quite important for physiological regulations. However, p-values are also helpful to explore, e.g., transcription factor binding sites. Moreover, amplitudes are taken into account in many studies (see e.g. papers of Naef, Korencic, Westermark, Ananthasubramaniam...). Since JTK or RAIN are non-parametric methods amplitudes are not in focus. The authors should discuss the biological relevance of amplitudes more clearly.

Thanks for raising this point. We are careful to limit our claims to bulk transcriptome data, and have tried to cite the relevant prior work. We have revised the Discussion to clarify what we see as the potential value of amplitudes, as illustrated by our analysis.

3. The selection of the 3 data sets and of specific genes seems reasonable since a range of technologies (microarrays versus RNS-seq), of durations (1 day versus 2 days), and of gene amplitudes are represented. Still the authors should comments their selections of data sets and genes.

We have added justification for our choices.

4. I find also the tissue-dependent phase distributions of clock-controlled genes of interest. However, a comparison with other studies (Zhang, GTEx from Talamanca et al.) and a discussion how amplitude thresholds such as 10%, 25%, 50% affect the phase distributions would be valuable.

Thank you for the suggestion. We initially explored several values of the amplitude threshold for those histograms (Figure S4C) before selecting the top 25%, all led to the same conclusion. We consider this a minor issue and tangential to the main point of the paper, so we have left the figure as is. We invite any interested reader to explore the publicly available results.

### Reviewer 3

#### Summary

The authors developed LimoRhyde2, a method for quantifying rhythmicity in genomic data, and applied it to mouse transcriptome data from liver, lung, and suprachiasmatic nucleus (SCN) tissues. The method uses periodic spline-based linear models and an Empirical Bayes procedure (Mash) to produce posterior fits and rhythm statistics. LimoRhyde2 prioritizes high-amplitude rhythms of various shapes rather than monotonic rhythms with high signal-to-noise ratios, which contrasts with previous methods like BooteJTK. The authors demonstrated the value of LimoRhyde2 in quantifying rhythmicity and highlighted some of its advantages over traditional methods. However, they also acknowledged limitations, such as the inability to compare rhythmicity between conditions and the assumption of fixed rhythms.

#### Major Comments

1. The key conclusions are convincing, as the authors demonstrated LimoRhyde2's ability to fit non-sinusoidal rhythms and prioritize high-amplitude rhythms over monotonic rhythms with high signal-to-noise ratios. This is shown by the comparison with BooteJTK, a popular method in the field, and by the analysis of real circadian transcriptome data from mouse tissues. However, the authors acknowledged some limitations that could impact the method's broader applicability.

Thank you.

2. Data and methods are presented in a reproducible manner, with detailed descriptions of the periodic spline-based linear models, the use of Mash for moderating raw fits, and the calculation of rhythm statistics. This information is sufficient for other researchers to replicate the study and apply the LimoRhyde2 method to their own datasets. The code is available already.

Thank you.

3. Adequate replication and statistical analysis are provided, with the authors analyzing the same datasets using both LimoRhyde2 and BooteJTK to compare their performance. The use of Spearman correlation to assess the relationship between the adjusted p-values from BooteJTK and the amplitudes from LimoRhyde2 further supports the statistical rigor of the study.

Thank you.

#### Minor Comments

1. Addressing LimoRhyde2's limitations would help improve the study.

We have extensively addressed the method’s limitations to the best of our knowledge in Discussion paragraphs 6 and 7.

2. Authors could provide more details on how LimoRhyde2 could be applied to single-cell RNA-seq data to improve the presentation. Single-cell quantification over time would be a challenging task, so some insight into this would be appreciated, rather than a brief comment at the end of the paper.

Thank you for your interest in this topic. To do it justice, however, requires its own project and paper, so scRNA-seq is beyond the scope of the current paper.

#### Significance Comments

1. This study represents a technical advance in the field of genomic analysis of biological rhythms by introducing LimoRhyde2, a method that prioritizes high-amplitude rhythms and directly estimates biological rhythms and their uncertainty. The method's ability to capture non-monotonic rhythms and account for uncertainty makes it a valuable tool for researchers interested in understanding circadian systems and their physiological impact.
2. The work is placed in the context of existing literature, as the authors compare LimoRhyde2 with BooteJTK, a refinement of the popular JTK_CYCLE method. The comparison highlights the differences in output, prioritization, and runtime, demonstrating LimoRhyde2's potential advantages over traditional methods in the field.
3. However, BooteJTK is relatively underused compared to many other methods, partly because of the difficulty and time required to run the analysis. The paper would be improved by comparing LimoRhyde2 to JTK_Cycle itself, as well as RAIN and ARSER. The latter are the most commonly used methods for rhythm detection, and thus the value of the paper's findings would be far greater by comparing to these methods. Like LimoRhyde2, they are also not resource-intensive to run.

Thanks for your feedback on this point, which is one we discussed at length amongst ourselves. In the end, we decided on BooteJTK because it seems to be the best performing version of the most common method. ARSER and RAIN are simply not the standard, and based on our interpretation of the evidence, not generally superior to JTK. If we had selected the vanilla JTK_Cycle, we felt a reviewer could discard our results by saying "well, they're comparing their method to a version of a method known to be flawed". Given our objective to highlight the differences between prioritization based on estimated effect size and prioritization based on p-value, we do not see the value of including additional methods in the analysis.

4. LimoRhyde2's ability to efficiently prioritize large effects with functional significance in the circadian system can provide valuable insights for these researchers and advance the understanding of biological rhythms. The LimoRhyde2 approach is different to conventional reliance on arbitrary p- or q-values, which are taken as almost sacrosanct in the field as a measure of a dataset's worth. LimoRhyde2 could thus help to change this false perception of how to rate a circadian rhythm, which has particularly been ushered in by a reliance on JTK_Cycle p- and q-values as the method of choice for assigning meaningfulness to rhythms. Unfortunately, JTK_Cycle is very conservative and is limited to detecting sinusoidal-type rhythms. LimoRhyde2 could overcome these limitations (as RAIN does too) if widely adopted. However, to do this, it must be compared to things like JTK_Cycle directly.

See reply to Significance Comment 3 above.
