# Supplementary material for: LimoRhyde2: Genomic analysis of biological rhythms based on effect sizes
Source: PLoS One. 2023 Dec 14;18(12):e0292089. doi: 10.1371/journal.pone.0292089 (PMC10721038; doi:10.1371/journal.pone.0292089)
Supplement: Attachment — Submitted filename: limorhyde2 rhythmicity response to reviewers - plos one 1.docx [file pone.0292089.s006.docx]

# Response to Reviewers

Please see our responses below and the highlighted revisions to the manuscript.

### Reviewer 1

The study by Obodo et al provides a novel method to estimate rhythmicity. This new approach is highly welcomed, and the authors convincingly show the limitation of estimating rhythmicity by a fixed threshold (p-value) of rhythmicity with or without amplitude estimation. LimoRhyde2 bases on effect sizes estimation instead of p values for rhythmicity. The manuscript has also received criticism and suggestions from the Review Commons, which the authors have addressed. Therefore, my comments focus not on the methodological aspect but on a user’s perspective.

1. The manuscript would be improved if the authors provided a simple table in which it is mentioned the pro and cons of LimoRhyde2 against established methods just as JTK, BooteJTK, RAIN, DryR, CircaN, etc.

Thank you. We have added a table comparing various methods.

2. There is an increased interest in the field of detecting ultradian rhythms. There are few tools properly established for this detection. The manuscript would improve if the authors provided more detail on how LimoRhyde2 can be used (if at all) to detect ultradian rhythms.

As we mentioned, LimoRhyde2’s periodic splines can potentially fit ultradian peaks within a timecourse having a period of around 24 h. As with any other method, a period of 24 h is arbitrary; the ability to reliably detect or quantify ultradian rhythms will depend on the sampling frequency and signal to noise in the data. We have added this point to the Discussion.

3. The authors heavily criticize using p- or q-values to establish rhythmicity. I do not intend to over-discuss this issue, but the authors focus on amplitude instead. However, several potential candidates can be excluded by selecting only high-amplitude rhythms. This could lead to the loss of several potentially rewarding targets for follow-up analysis. Mild or low amplitude rhythmic genes can still show robust rhythms at the protein level and vice-versa. They should discuss this in brief.

We agree. There is no free lunch and no perfect method. Based only on transcriptome data, it’s hard to know what the levels of the protein or any of its post-translational modifications are doing. Our objective is to provide a practical method for estimating effect sizes and to highlight the advantages of considering them in a rhythmicity analysis. We now state more explicitly in the Discussion that low-amplitude rhythms could still be biologically meaningful.

4. In addition to amplitude, phase is an essential parameter for circadian studies. Phase assessment is not discussed in the manuscript. The manuscript would improve if the authors provided some evidence for phase assessment compared to established methods.

Thanks for bringing this up. The phase estimates of BooteJTK and LimoRhyde2 are highly correlated, which we have added to the Results.

5. Recently Brooks et al (10.1177/07487304231179600) showed concerning findings on the lack of reproducibility of circadian/diurnal transcriptome across several studies. In particular, the authors only focused on phase as amplitude was highly variable across the studies. Considering that LimoRhyde2 method is based on amplitude estimation, how trustworthy would this method be to assess rhythmicity across similar studies? The authors should discuss this in the manuscript.

We have a somewhat different interpretation of Brooks et al.’s results, which showed generally low overlap, between studies, of genes called rhythmic (according to JTK). It seems unlikely that any statistical method will resolve this poor reproducibility. In any case, expression values are not necessarily comparable between studies (even if both use RNA-seq), so neither are amplitudes *in the original units*. Perhaps meta-analyses based on LimoRhyde2-derived amplitudes could compare percentiles or otherwise scaled values.

### Reviewer 2

This revised manuscript addressed the issues raised by the previous 3 reviewers and described an interesting new method to characterize rhythmicity of gene expression.

1. The limitations of the method are well described in the discussion. Nevertheless, one limitation still needs to be addressed. As stated by the authors in the introduction, many methods rely on timepoints being equally spaced and cannot cope with missing data. However, authors did not provide evidence that LimoRhyde2 can perform well in such conditions and even replaced missing values by “non-zero counts” from other time points in the chosen mouse datasets. Therefore, it could be great if the authors could expand their validation (for example using simulated data) by testing alternative sampling frequencies (2 hour, 4 hour, 6 hour), sampling durations (1 cycle vs 2 cycles, vs partial cycle), replicate numbers, and in datasets with missing timepoints/values.

We agree it is important to test the method on different experimental designs, which is why we selected three such different datasets. As mentioned in the Discussion, we deliberately focused on real data instead of simulated data, and we do not plan to exhaustively test every combination of experimental parameters. Importantly, LimoRhyde2 does not replace missing values; as it simply fits a linear model, it would ignore missing values and smoothly handle unevenly spaced timepoints.

The zero counts in the RNA-seq datasets are not exactly missing, they are estimated as zero. To ignore them completely would bias the estimated expression of that gene at that timepoint, which is why we perform the simple imputation described in the Methods, which simply provides a more realistic lower bound for the gene’s expression level.

### Reviewer 3

I am happy with authors' response to my comment in the first round (via Review Commons). I do not have any additional comments for the authors. Thank you for taking the time to address my previous concerns.

Thank you for the comment.
